# N-P Fertilization Inhibits Growth of Root Hemiparasite *Pedicularis kansuensis* in Natural Grassland

**DOI:** 10.3389/fpls.2017.02088

**Published:** 2017-12-07

**Authors:** Yanyan Liu, Teyibai Taxipulati, Yanming Gong, Xiaolin Sui, Xuezhao Wang, Serge-Étienne Parent, Yukun Hu, Kaiyun Guan, Airong Li

**Affiliations:** ^1^Key Laboratory of Biogeography and Bioresource in Arid Land, Xinjiang Institute of Ecology and Geography, Chinese Academy of Sciences, Ürümqi, China; ^2^College of Resources and Environmental Science, Xinjiang University, Ürümqi, China; ^3^Yunnan Key Laboratory for Wild Plant Resources, Department of Economic Plants and Biotechnology, Kunming Institute of Botany, Chinese Academy of Sciences, Kunming, China; ^4^College of Animal Science and Technology, Northwest Agriculture and Forestry University, Xianyang, China; ^5^Department of Soils and Agri-Food Engineering, Université Laval, Québec, QC, Canada

**Keywords:** fertilization, grassland, root hemiparasite, plant functional groups, foliar nutrient balance

## Abstract

Fertilization has been shown to affect interactions between root hemiparasitic plants and their host plants, alleviating damage to the hosts by parasitism. However, as a majority of studies were conducted in pot cultivation, the influence of fertilizer application on root hemiparasites and the surrounding plant community in field conditions as well as relevant mechanisms remain unclear. We manipulated soil nutrient resources in a semi-arid subalpine grassland in the Tianshan Mountains, northwestern China, to explore the links between fertilization and plant community composition, productivity, survival, and growth of a weedy root hemiparasite (*Pedicularis kansuensis*). Nitrogen (at a low rate, LN, 30 kg N ha^-1^ year^-1^ as urea; or at a high rate, HN, 90 kg N ha^-1^ year^-1^ as urea) and phosphorus [100 kg ha^-1^ year^-1^ as Ca(H_2_PO_4_)_2_⋅H_2_O] were added during two growing seasons. Patterns of foliar nutrient balances were described with isometric log ratios for the different plant functional groups receiving these fertilization regimes. Fertilization with LN, HN, and P reduced above-ground biomass of *P. kansuensis*, with above-ground biomass in the fertilization treatments, respectively, 12, 1, and 39% of the value found in the unfertilized control. Up to three times more above-ground biomass was produced in graminoids receiving fertilizers, whereas forb above-ground biomass was virtually unchanged by the fertilization regimes and forb species richness was reduced by 52% in the HN treatment. Fertilization altered foliar nutrient balances, and distinct patterns emerged for each plant functional group. Foliar [C | P,N] balance in the plant community was negatively correlated with above-ground biomass (*P* = 0.03). The inhibited competitiveness of *P. kansuensis*, which showed a much higher [C | P,N] balance, could be attributed to reduced C assimilation rather than mineral nutrient acquisition, as shown by significant increase in foliar N and P concentrations but little increase in C concentration following fertilization.

## Introduction

Root hemiparasitic plants are green plants with retained photosynthetic capability, but still partially depend on a host plant for water, mineral nutrients and in many cases carbohydrate supply (Press, 1995; Irving and Cameron, 2009; Těšitel et al., 2011). These plants often take up substantial amount of nutrients from host plants, leading to significant suppression of host growth and reduction in host productivity (Press and Phoenix, 2005). Furthermore, root hemiparasitic plants can alter plant community structure and plant diversity by changing competitive relationships between host and non-host plant species (Hedberg et al., 2005; Borowicz and Armstrong, 2012; Bao et al., 2015), causing significant effects on above and below-ground ecosystems at various trophic levels (Bardgett et al., 2006; Hartley et al., 2015). Despite their ubiquity in territorial ecosystems (Press, 1995) and being long considered as keystone species (Press and Phoenix, 2005), investigations of root hemiparasitic plants under field conditions are limited when compared with numerous studies of other plant species. This is particularly the case for root hemiparasites occurring in natural or semi-natural ecosystems. Apart from *Striga* species, which cause large yield loss to crops in Africa (Simier et al., 2006), only a very limited number of root hemiparasites (such as *Rhinanthus*) have been extensively studied (Fisher et al., 2013; Hartley et al., 2015; Těšitel et al., 2017). How other root hemiparasites interact with the surrounding plant community under various environmental conditions remains unexplored.

Previous studies suggest that root hemiparasitic plants often occur in low-nutrient soils (Press and Phoenix, 2005), probably due to relatively weak competition for light and space compared to their host plants. Fertilizer application has been shown to cause seedling mortality (Mudrak and Leps, 2010) or suppressed growth in root hemiparasites (Gibson and Watkinson, 1991; Davies and Graves, 2000; Borowicz and Armstrong, 2012), thus alleviating damages to host plants by the hemiparasites. As a result, fertilization was suggested to be an effective practice to control parasitic weeds (Eplee and Norris, 1995; Davies and Graves, 2000). However, growth response to different nutrients varied greatly among root hemiparasites (Davies and Graves, 2000), even between closely related species (Li et al., 2013). In addition, other factors such as host identity could influence the outcomes of fertilization (De Hulla, 1985).

*Pedicularis kansuensis* is an annual or biennial root hemiparasite commonly found in subalpine zone of western China. This fast spreading (estimated spread rate of 3.3 × 10^3^ ha year^-1^; Liu et al., 2008) hemiparasite has become a severe problem in many regions over the past decade, reducing forage crop productivity in grasslands up to 80% and threatening the local livestock industry (Li et al., 2006; Liu et al., 2008; Bao et al., 2015; Sui et al., 2015). So far, no effective control strategy has been identified for this root hemiparasite. Because the infestation of root hemiparasites was suggested to be influenced by soil nutrient levels (Borowicz and Armstrong, 2012), it is relevant to test if application of fertilizers could alleviate the problem. According to a field survey report (Bao et al., 2015), *P. kansuensis* often reduce the above-ground biomass of graminoids and in some cases of legumes, but had little influence on forb biomass. We therefore presumed that fertilization could benefit more to graminoids than forbs if the infestation of *P. kansuensis* must be alleviated.

Because plant parasitism is a strategy evolved for nutrient and water acquisition, the interactions between root hemiparasites and the surrounding plant community under different fertilization regimes may be reflected by changes in their tissue nutrient composition. Although nutrient stoichiometry has been considered an important controlling factor for performance of root hemiparasitic plants (Quested et al., 2002), its significance in shaping grassland communities where root hemiparasites occur in large abundance has been scarcely addressed.

Plant nutritional issues are often diagnosed using concentrations of nutrients and/or nutrient ratios such as the well known nitrogen to phosphorus ratio (N:P) (Braakhekke and Hooftman, 1999; Güsewell, 2004; Peñuelas et al., 2012; Di Palo and Fomara, 2017). However, dual ratios provide a limited view of the plant nutrient-acquisition system and cannot fully reflect nutrient interactions. The ratios can be arranged in numerous manners, exceeding the degrees of freedom of a vector of concentrations (Parent et al., 2012). Moreover, the compositional nature of tissue concentration data makes direct interpretation of the raw data or dual ratios problematic without proper transformation, often leading to conflicting results and wrong inferences (Parent et al., 2013a). Recently, a balance diagnostic approach using the isometric log-ratio (*ilr*) data transformation has been proposed in plant nutritional studies (Parent et al., 2013b; Nowaki et al., 2017). This approach uses orthonormal binary nutrient partitions instead of dual ratios to account for elemental nutrient signatures (ionomes), thus overcoming the flaws in previous methods and allowing an unbiased interpretation of nutrient profiles. Because many root hemiparasites have much higher tissue N and P concentrations (Quested et al., 2002; Li et al., 2013) than their hosts but assimilate much less C (Phoenix and Press, 2005), fertilization alter differentially the N-P-C balances of plant functional groups involving a root hemiparasite. Fertilization often increases competition between plants for light (Aerts, 1999), where in most cases root hemiparasites are at a disadvantage (Těšitel et al., 2013). We therefore presumed that fertilization could inhibit the competitiveness of *P. kansuensis* by reducing C assimilation, causing the N-P-C balances to shift away from the C side. To our knowledge, no field investigation has been carried out to investigate the influence of fertilization on a root hemiparasite and the surrounding plant community from the perspective of tissue nutrient balances.

The objective of this study was to test the influence of N and P fertilization on plant abundance, canopy cover, biomass, and C-N-P balances in *P. kansuensis* as well as other plant functional groups in Bayanbulak Grassland, China. The following hypotheses were tested: (1) Fertilization reduces *P. kansuensis* abundance and biomass in the plant community; (2) Fertilization increases graminoid biomass and abundance in the plant community, but has no effect on forbs; (3) Fertilization alters plant foliar nutrient balances, but different functional groups show different patterns, with the N-P-C balances leaning toward the C side in graminoids but the opposite side in *P. kansuensis.*

## Materials and Methods

### Study Area

This study was carried out near the Bayanbulak Grassland Ecosystem Research Station, Chinese Academy of Sciences (42°53.1′N, 83°42.5′E). Located in Bayanbulak Basin of the Tianshan Mountains in northwest China, Bayanbulak Grassland is the second largest grassland in China, covering an area of approximately 23,000 km^2^. The grassland is natural grassland for grazing in spring and autumn (Yerdaolaiti, 1989). Mean altitude is 2500 m (Gong et al., 2010). Meteorological conditions are presented in **Table 1** (data provided by Bayanbulak, Tianshan Mountains. meteorological station). Mean annual precipitation is 300.8 mm, 77.6% occurring from May to August. Mean annual temperature is -4.8°C, being lowest in January (-26.7°C) and highest in July (11.6°C).

**Table 1:** Data on precipitation (mm) and temperature (°C) in the study area (provided by the Meteorological Station of Bayanbulak, Tianshan Mountains).

Year	Precipitation (mm)					Mean Temp. (°C)	
		
	May	June	July	August	Total (May to August)	January	July
2010	21.7	103.4	95.9	44.3	265.3	-22.7	12
2011	29.5	90.5	44.7	111.1	275.8	-33.6	11.5
2012	15	46.8	116.1	32.1	210	-25.4	11.2
2013	8	76	59.9	73.1	217	-28.1	10.6
2014	20.4	68.5	80.8	20	189.7	-27.2	10.6
2015	69.3	71.3	32.6	70.2	243.4	-23.3	13.9


The soil is a Cambisol in the Food and Agriculture Organization (FAO) soil classification system, high in organic matter and nitrogen, and low in phosphorus (**Table 2**). According to previous studies carried out in this area (Gong et al., 2010), there are 36 plant species belonging to 26 genera and 16 families, with *Festuca ovina* and *Stipa purpurea* as dominant species.

**Table 2:** Soil nutrient profiles in the research area sampled 2 years after fertilizer application.

Soil depth	Treatment	pH	Soil organic matter g kg^-1^	Total N g kg^-1^	Total P g kg^-1^	Sum of nitrate and ammonium N mg kg^-1^	Olsen P mg kg^-1^
0–10 cm	CK	7.69	80.13	3.41	2.32	65.19	38.60
	LN	7.74	76.48	4.03	2.54	65.63	16.60
	HN	7.73	67.25	3.03	2.48	**79.19**	28.72
	P	7.41	80.96	3.00	2.65	66.94	**175.55**
10–20 cm	CK	8.13	56.64	2.81	2.20	44.63	21.38
	LN	8.09	56.63	2.46	2.33	38.94	17.44
	HN	8.03	56.43	3.33	2.36	37.63	17.86
	P	7.85	55.30	3.11	2.37	43.31	**42.91**
20–30 cm	CK	8.08	36.91	2.86	2.21	20.13	14.11
	LN	8.18	38.53	2.71	2.15	24.06	18.36
	HN	8.25	38.33	2.78	2.01	22.31	15.00
	P	8.11	38.50	2.41	2.30	27.13	**24.80**


*Values are presented as means of four replicates (bold fonts indicate significant increase at *P* < 0.05).*

### Experimental Design

In 2013, one permanent study site (100 m × 100 m) was established near the Bayanbulak Grassland Ecosystem Research Station where *P. kansuensis* infestation was heavy (canopy cover of the hemiparasite >20%). Sixteen 4 m by 3 m blocks were randomly assigned in early May 2013 to conduct fertilization tests. Plant community structure, total canopy cover and *P. kansuensis* abundance were similar across the study site.

Four fertilization regimes comprised: (1) N was applied at 30 kg N ha^-1^ year^-1^ as urea, a fertilizer widely used by local farmers, based on the average N dosage in Xinjiang arid land (LN), (2) N applied at 90 kg N ha^-1^ year^-1^ as urea, which is the highest N dosage in farmland ecosystem of North China (HN), (3) P applied at 100 kg Ca(H_2_PO_4_)_2_⋅H_2_O ha^-1^ year^-1^, a dosage suggested to stimulate plant biomass production in low-P soils (Tilman, 1988), and (4) control without fertilizer application (CK). Each treatment was replicated four times in a completely randomized design. A 2 m buffer zone was maintained between blocks. Fertilization treatments were divided into two equal parts to reduce ammonia volatilization from urea and thus enhance fertilization efficiency, being applied in mid-June (seeding and juvenile stage for *P. kansuensis*) and mid-July (branching period for *P. kansuensis*) in 2014 and 2015, respectively. Fertilizers were mixed with 1 kg soil to facilitate application. The control blocks received the same amount of soil but without fertilizer.

### Sampling and Data Collection

Before fertilizer application, a background survey was conducted in early September, 2013. Plant species composition, species number, abundance, canopy cover, as well as above and below-ground biomass per unit area were recorded in each block based on plant functional groups (graminoids, legumes, forbs, and the root hemiparasite).

In each block, two 1 m by 1 m plots were randomly assigned to monitor the effects of fertilization on the parameters recorded before fertilization. One plot was surveyed in early September 2014 and another one in early September 2015. The aboveground vegetation in the 1 m by 1 m plots was clipped at a height of 2 cm, sorted into functional groups (graminoids, legumes, forbs, and *P. kansuensis*), dried at 65°C for 48 h, and weighed. A soil auger (8 cm in diameter and 10 cm in depth; P. O. Box 4-6987 ZG Glesbeek, The Netherlands) was used to sample the roots and determine below-ground biomass. Three soil cores per plot were taken at 0–10, 10–20, and 20–30 cm soil depths, respectively. Roots were extracted from the soil core, dried at 65°C for 48 h, and weighed. Because it was impossible to separate root systems from different plant groups, root material was pooled rather than divided into functional groups. Soil samples from the same depth were pooled together after root extraction for nutrient analysis. Plant canopy cover, defined as the proportion of the ground occupied by a perpendicular projection of the aerial parts of individuals, was estimated by eye for each functional group. Plant abundance was the number of individuals per square meter. Plant community composition was measured as species richness (number of plant species) and canopy cover of different functional groups.

### Soil Nutrient Analysis

Soil samples were dried at room temperature and sieved less than 1 mm. Soil pH was measured from a 1:5 soil:H_2_O mixture using a pH meter (Mettler–Toledo 320, Mettler Toledo Instruments Co. Ltd., Greifensee, Switzerland). Soil organic matter was determined using a modified Walkley-Black chromic acid wet oxidation method (Wang et al., 2012). A fraction of the samples were ground to pass through a 0.1 mm sieve for total N and total P determination. Total N was determined by the modified semimicro-Kjeldahl method (Liao, 1981), and total P by the NaOH digestion and quantified by colorimetry (Smith and Bain, 1982). The plant-available N (sum of nitrate and ammonium N) was measured in 2 M KCl soil extracts using a Lachat Flow Injection Analyzer Quikchem 8500 S2 (Lachat Instruments, Hach Company, United States). Plant available P was reported as Olsen-P (Olsen et al., 1954).

### Plant Tissue Nutrient Analysis

Dried leaves were milled (Retsch MM 400, Retsch GmbH and Co. KG, Haan, Germany) to less than 1 mm. Total carbon and total N concentration were quantified using an elemental analyzer NA1500 (Carlo Erba, Thermo Fisher Scientific, United States). Foliar P concentration was determined using the molybdate/stannous chloride method (Kuo, 1996), after digesting about 0.1 g sample in H_2_SO_4_-H_2_O_2_ (Bennett et al., 2003). All concentrations were calculated on a dry mass basis.

### Statistical Analysis

One-way ANOVA was used to analyze plant canopy cover, above-ground biomass, below-ground biomass, plant abundance, species richness, and foliar nutrient (C, N, P) concentrations. Linear regression models were used to explore the relationship between above-ground biomass of *P. kansuensis* and that of other plant functional groups as well as ln-transformed below-ground biomass. One-way repeated ANOVA was used to test the year-to-year variations in species richness, *P. kansuensis* abundance, canopy cover and above-ground biomass. Data were checked for normal distribution. Significance level was 0.05. Most statistical analyses were carried out using Origin 8.0 (ORIGINLAB Co., Northampton, MA, United States). Graphs were drawn using SigmaPlot (SigmaPlot for Windows, Version 10, SyStat Software Inc., San Jose, CA, United States).

Nutrient balances were calculated with the isometric log-ratio (*ilr*) method (Parent et al., 2013b) in foliar tissues of plant functional groups (graminoids, legumes, forbs, and *P. kansuensis*). We designed three orthonormal balances based on prior and expert knowledge on nutrient interactions. Nutrients were first contrasted with the filling value (Fv) computed by difference between unit of measurement and the sum on nutrient concentrations, referred to as [Fv | C,P,N]. A second balance contrasted C with P and N as [C | P,N]. A third balance contrasted P and N as [P | N]. By convention, nutrients on the left of the vertical bar are denominators of the isometric log-ratios, while nutrients on the right of the vertical bar are numerators. Nutrient compositions were transformed into *ilr* using the R compositions package (van den Boogaart et al., 2014).

Statistical computations were conducted in the R statistical environment (R Core Team, 2017). To test if the patterns of foliar nutrient balances were influenced by fertilization regimes (LN, HN, P, and unfertilized CK) or plant functional groups (graminoids, legumes, forbs, and *P. kansuensis*), we analyzed the fertilization experiment as linear mixed-effect models on each balance with random effect on the intercept by year using the R nlme package (Pinheiro et al., 2017), with fertilization regimes and plant functional groups as fixed effects and year of survey as a random effect. Data from unfertilized control blocks were used as a reference for fertilization and graminoids data were used as a reference for other plant functional groups. We tested the difference between other factors and the reference. To test if the nutrient balance correlated with above-ground biomass, we analyzed the effect of *ilr* on the log of above-ground biomass with a linear mixed-effects model with a random effect on the intercept by year of study.

## Results

### Effects of Fertilization on Soil Nutrient Profiles

The N fertilization showed negligible effect on total soil N, but slightly increased available mineral N in 0–10 cm when applied at a higher rate (**Table 2**). Soil available P was greatly increased by P fertilizer at all soil depths, with the greatest increase (about five times that of the control) in the top 10 cm.

### Effects of Fertilization on *P. kansuensis* Abundance and Above-Ground Biomass

**FIGURE 1:**
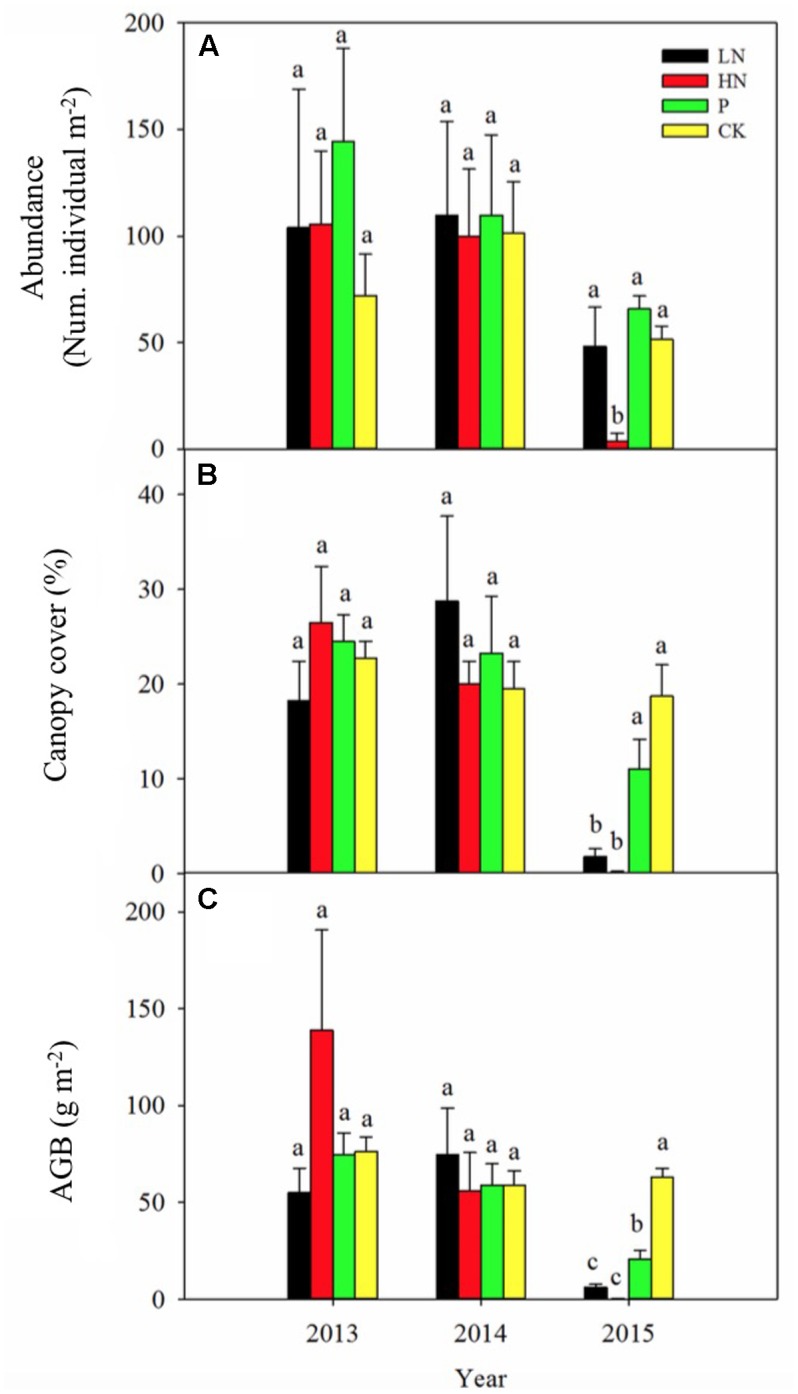
Plant abundance **(A)**, canopy cover **(B)** and above-ground biomass (AGB; **C**) of *Pedicularis kansuensis* under various fertilization regimes before (2013) and after (2014 and 2015) fertilization. Different letters indicate significant differences between treatments within the same year at *P* < 0.05, *n* = 4. Values are presented as means ± SE. Fertilization regimes: CK, unfertilized control; LN, fertilized with lower nitrogen level; HN, fertilized with higher nitrogen level; P, fertilized with phosphorus.

Before fertilization, *P. kansuensis* abundance (106 individuals per square meter on average, *n* = 16), canopy cover (23% on average, *n* = 16) and above-ground biomass (86 g dry biomass per square meter on average, *n* = 16) were similar (with no statistical significance) among blocks (**Figure 1**). Year-to-year variations in the measured parameters were of no statistical significance, as shown in the control blocks.

Fertilization showed no significant influence on plant abundance, canopy cover or above-ground biomass of *P. kansuensis* during the first year of application (**Figure 1**). However, abundance of *P. kansuensis* declined by 92% (*P* < 0.05) in HN treatment (4 plants per square meter on average) during the second year compared with the unfertilized control (51 plants per square meter on average) (**Figure 1A**). Plant canopy cover and above-ground biomass of *P. kansuensis* were significantly reduced by 91 and 88% in LN (*P* < 0.05) and 99 and 99% in HN (*P* < 0.05). Although abundance and canopy cover of *P. kansuensis* was not affected by P fertilization, a significant decline by 61% in above-ground biomass (*P* < 0.05) was detected in the second year of P fertilization.

### Effects of Fertilization on Plant Community Composition

**FIGURE 2:**
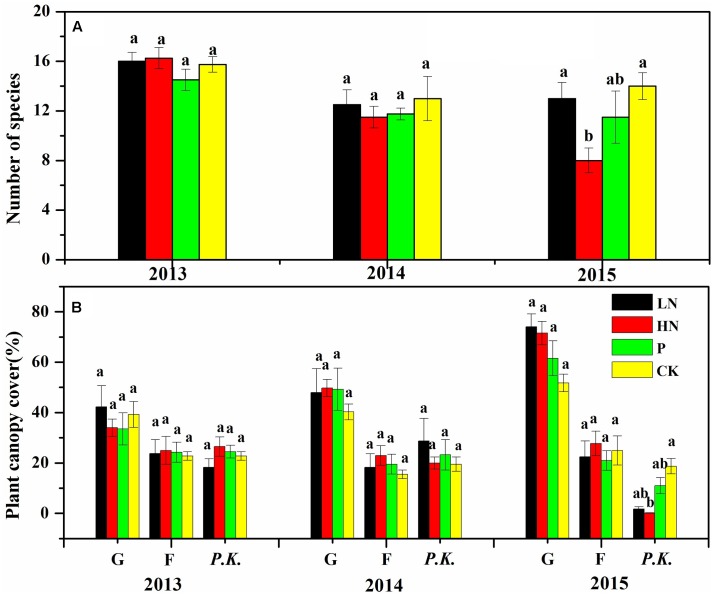
Species richness **(A)** and plant canopy cover of different plant functional groups **(B)** under various fertilization regimes before (2013) and after (2014 and 2015) fertilization. Different letters indicate significant differences between treatments within the same year at *P* < 0.05, *n* = 4. Values are presented as means ± SE. G, graminoid plants; F, forbs; *P.K.*, *P. kansuensis*. Fertilization regimes: CK, unfertilized control; LN, fertilized with lower nitrogen level; HN, fertilized with higher nitrogen level; P, fertilized with phosphorus.

**FIGURE 3:**
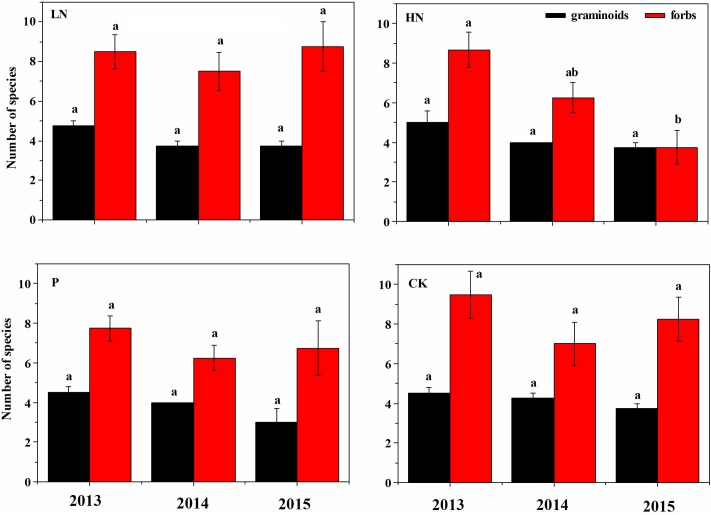
Number of graminoids and forbs species under various fertilization regimes before (2013) and after (2014 and 2015) fertilization. Different letter indicates significant difference between years of observation at *P* < 0.05, *n* = 4. Values are presented as means ± SE. Fertilization regimes: CK, unfertilized control; LN, fertilized with lower nitrogen level; HN, fertilized with higher nitrogen level; P, fertilized with phosphorus.

Before fertilization, plant species richness and canopy cover of different plant functional groups were comparable among blocks (**Figure 2**). Plant species richness was not affected by fertilizer treatment during the first growing season, but significantly reduced by 51% in HN treatment (*P* < 0.05) during the second growing season compared to the unfertilized control (**Figure 2A**). A majority of reduced plant species were forbs, as shown by the decline by 52% (*P* < 0.05) in species number of forbs after HN treatment compared to the unfertilized control (**Figure 3**).

Overall, canopy cover of graminoids showed much stronger response than forbs to N fertilization. After 2 years of N fertilizer application, canopy cover of graminoids almost doubled in N-fertilized treatments (LN and HN), while forbs canopy cover showed little response (**Figure 2B**). Canopy cover of *P. kansuensis* was significantly reduced in the N-fertilized treatments following 2 years of application.

### Effects of Fertilization on Plant Community Productivity

**FIGURE 4:**
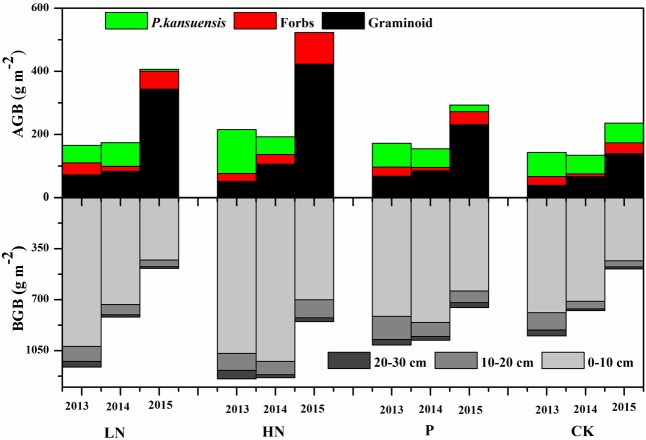
Above-ground biomass and below-ground biomass (BGB) of different functional groups under various fertilization regimes before (2013) and after (2014 and 2015) fertilization. Values are presented as means of four replicates. Fertilization regimes: CK, unfertilized control; LN, fertilized with lower nitrogen level; HN, fertilized with higher nitrogen level; P, fertilized with phosphorus.

Before fertilizer application, the blocks had similar above-ground biomass and below-ground biomass, except that the blocks assigned to HN showed much higher *P. kansuensis* biomass (**Figure 4**). In 2015, all blocks produced higher above-ground biomass than the previous years. The fertilized (particularly N fertilized) treatments showed much larger increase in above-ground biomass than control.

The total above-ground biomass was not influenced by fertilization in the first year of application, but was greatly increased in the second year (**Figure 4**). The increase in total above-ground biomass was mainly due to an increase in graminoids biomass. Although total above-ground biomass showed little change in response to fertilization in the first growing season following fertilization, *P. kansuensis* biomass was reduced by more than two thirds in the HN plots, and graminoids biomass increased. In the second year of fertilization, graminoids biomass was greatly increased and *P. kansuensis* biomass decreased in response to fertilization. In N treatments, graminoids above-ground biomass increased more than three times while only very limited amount of *P. kansuensis* biomass was produced. Across 2 years of fertilization, above-ground biomass proportion of *P. kansuensis* dropped from 36 to 47% in average before fertilization application to 1.51% in LN treatment, 0.04% in HN treatment, and 6.97% in P treatment.

Plant roots were distributed mainly in the top 0–10 cm, accounting for more than 82% of the root biomass in most cases. Root biomass in 10–20 cm and 20–30 cm layers accounted for 15 and 5% of below-ground biomass, respectively. As above-ground biomass increased, below-ground biomass decreased. However, no clear pattern was observed in terms of below-ground biomass response to fertilization.

**FIGURE 5:**
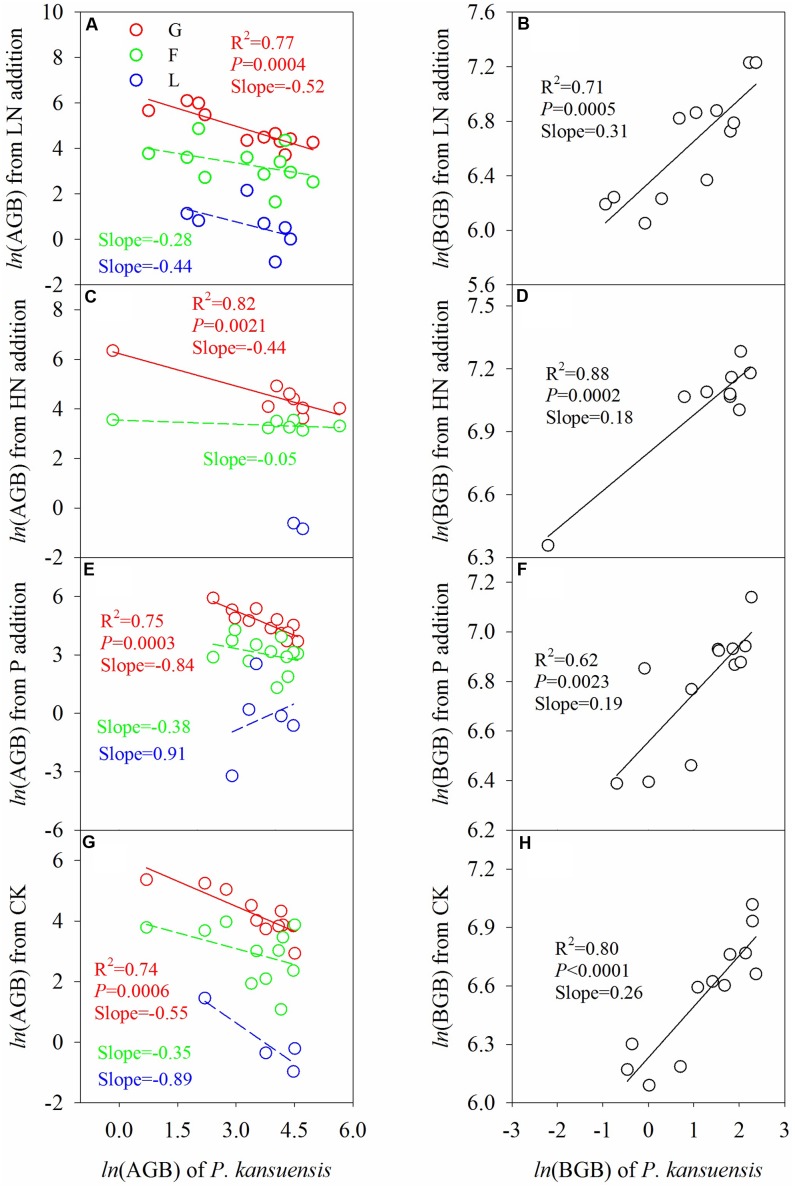
Relationship between *P. kansuensis* and other plant functional groups in terms of AGB **(A,C,E,G)** and BGB **(B,D,F,H)** in the second growing season after fertilization. Total BGB of other functional groups from the same soil core were pooled together for analysis, as it was not possible to separate the roots. Data was log-transformed (*ln*) before analysis to meet the assumptions of the analysis. G, graminoids; F, forbs; L, legumes. Fertilization regimes: CK, unfertilized control; LN, fertilized with lower nitrogen level; HN, fertilized with higher nitrogen level; P, fertilized with phosphorus.

Across fertilization regimes, above-ground biomass of *P. kansuensis* correlated negatively with that of graminoids, but not that of forbs (**Figure 5**). There was a positive correlation between below-ground biomass of *P. kansuensis* and below-ground biomass of the plant community. Fertilization mitigated the negative effect of *P. kansuensis* on above-ground biomass of graminoids, especially in HN treatment.

### Effects of Fertilization on Foliar Nutrient Profiles

**FIGURE 6:**
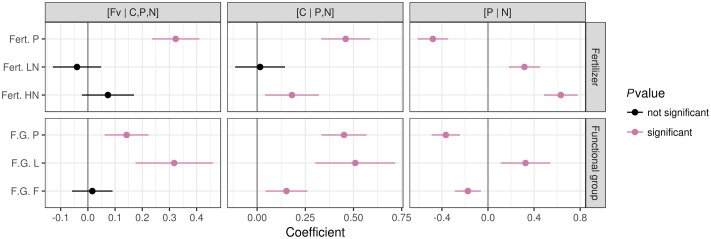
Patterns of foliar nutrient balances under different fertilization regimes and in different functional plant groups. Data from unfertilized blocks were used as a reference for fertilization treatments. Data from graminoids were used as a reference for other plant functional groups. The significance was tested at *P* < 0.05. Notes: *Fert. P*, fertilized with P at a rate of 100 kg Ca(H_2_PO_4_)_2_ ha^-1^; *Fert. LN*, fertilized with low N level at a rate of 30 kg N ha^-1^ as urea; *Fert. HN*, fertilized with high N level at a rate of 90 kg N ha^-1^ as urea; *F.G.*, functional plant groups; *P*, *Pedicularis kansuensis*; *L*, legumes; *F*, forbs.

**FIGURE 7:**
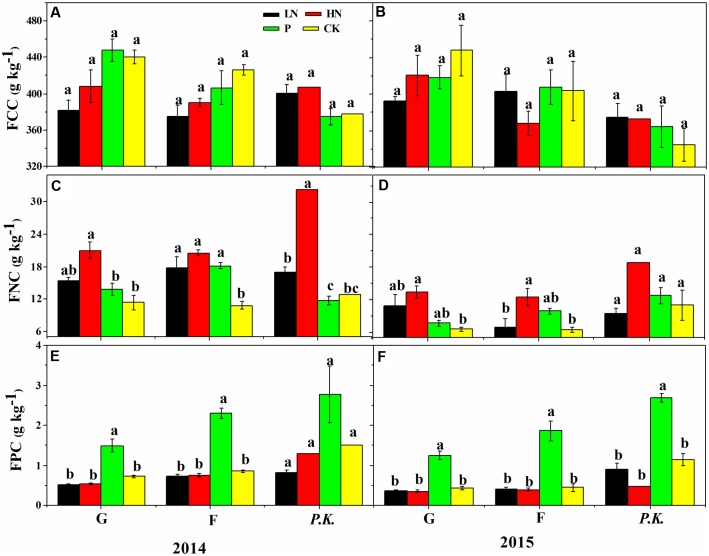
Foliar element concentrations of different plant groups under different nutrient supply regimes in the first (2014) and second (2015) growing season after fertilization. **(A,B)** Foliar carbon concentration (FCC, g kg^-1^); **(C,D)** foliar nitrogen concentration (FNC, g kg^-1^); **(E,F)** foliar phosphorous concentration (FPC, g kg^-1^). G, graminoids; F, forbs; P.K., *P. kansuensis*. Data are presented as means ± SE of four replicates. Bars with different letters indicate significant difference at *P* < 0.05 level. Fertilization regimes: CK, non-fertilized control; LN, fertilized with lower nitrogen level; HN, fertilized with higher nitrogen level; P, fertilized with phosphorus.

Compared to control, P fertilization significantly (*P* ≤ 0.05) altered plant nutrient balances (**Figure 6**) with a positive effect on [Fv | C,P,N] and [C | P,N], and a negative effect on [P | N]. In all cases, P fertilization shifted plant nutrient balances toward P due to an increase in foliar P concentration (**Figure 7**).

The N fertilization significantly shifted [P | N] toward N (**Figure 6**) by increasing foliar N concentrations (**Figure 7**), but showed little influence on the [Fv | C,P,N] balance. The [C | P,N] balance leaned toward the [P,N] side with HN, due to higher foliar N concentrations under HN than LN.

**FIGURE 8:**
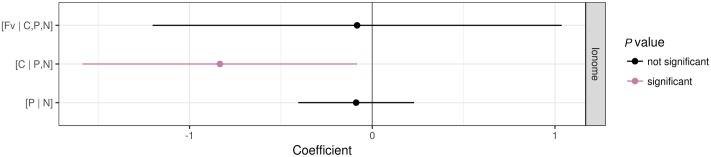
Coefficients of a linear mixed model showing the effect of nutrient balances on the log of plant AGB. The significance was tested at *P* < 0.05.

Patterns of foliar nutrient balances varied greatly among plant functional groups (**Figure 6**). In contrast to graminoids, other functional groups showed higher (*P* ≤ 0.05) [C | P,N] balances due to slightly higher C concentrations but much lower P and N concentrations in graminoids than other groups (particularly *P. kansuensis* in fertilized treatments; see **Figure 7**). In control, foliar C concentration of *P. kansuensis* was half that of surrounding plants but had about twice as much N and P concentration despite N concentration comparable to the surrounding plants during the first growing season. Fertilization significantly increased foliar N and P concentrations in *P. kansuensis* and increased C concentrations little, resulting in significant increase in the [C | P,N] balance. Year-to-year variations in plant elemental concentrations did not influence foliar nutrient balances significantly. Only the [C | P,N] balance influenced above-ground biomass (coefficient = -0.8342, *P* = 0.03; **Figure 8**). Plants showing lower [C | P,N] balance (higher C concentrations or lower N and P concentrations) tended to produce higher above-ground biomass.

## Discussion

### Fertilization Reduced Abundance and Growth of *P. kansuensis*

Application of N and P fertilizers reduced plant abundance and above-ground biomass of *P. kansuensis*. The significant decline in plant abundance and above-ground biomass of the root hemiparasite after fertilization (particularly a higher rate of N fertilization) agreed with previous reports on fertilizer-induced growth suppression in root hemiparasites (Mudrak and Leps, 2010; Borowicz and Armstrong, 2012). The N fertilization can impair the survival and development of plant species, presumably as a result of P deficiency or nutritional imbalance with other elements (Güsewell, 2004). In addition, increased N load may have direct toxic effects on some root hemiparasitic plants due to excess ammonium accumulation, as reported for *Striga hermonthica* under high N levels (Simier et al., 2006). Apart from direct fertilization effect, shading by the vigorous growth of graminoids may be an important factor reducing the abundance and growth of *P. kansuensis*. Higher mortality rate or suppressed growth of root hemiparasitic plants occurred when grown with vigorous hosts competing for light (Matthies, 1995; Těšitel et al., 2013). In *Pedicularis* species, imbalanced nutrient supply (Li et al., 2013) and competition for light from vigorous growth of hosts (Li et al., 2012; Mardoian and Borowicz, 2016) were found to reduce above-ground biomass or survival of the hemiparasite. However, a complete separation of fertilizer effect and shading effect is not possible from this field experiment, because of confounding effects of nutrient availability on plant productivity (shading effects). Cultivation experiments on *P. kansuensis* separating the effect of nutrient supply and that of shading are required to determine if it is nutritional imbalance, toxic overload, shading, or a combination of all the mechanisms that account for the inhibited competitiveness of the hemiparasite following fertilization.

In comparison with the results obtained in pot cultivation, where N effects on root hemiparasite was significant after just a few months, it took a longer period under field conditions, suggesting high buffering capacity of the ecosystem. N losses through volatilization, indicated by little N accumulation in the soil when fertilized with urea, may partially account for the time lag. We expect to see more significant effects of fertilization over extended experimental duration. Because the soil was alkaline in the research area, urea applied onto the soil surface could rapidly transform into volatile ammonia by ubiquitous enzyme urease (Gioacchini et al., 2002), which may have toxic effects on the seedlings (Bremner and Krogmeier, 1989; Britto and Kronzucker, 2002). Further studies are required to set apart the toxic effect of ammonia from that of nitrogen fertilization on plant abundance of the hemiparasite.

### Fertilization Was More Beneficial to Graminoids than Forbs and Altered Plant Community Composition

As expected, graminoids benefited more from fertilization than forbs, as shown by significant increase in above-ground biomass over control. By contrast, forb above-ground biomass hardly increased by any fertilization regimes, while forb species richness was reduced by 52% in HN blocks after 2 years of fertilization. It has been suggested that balanced resource supply would be required to reach high plant species diversity (Braakhekke and Hooftman, 1999). Imbalanced nutrient input (particularly excess N) has been considered the main cause for species loss in grassland ecosystems, most likely by hindering the acquisition of other elements (Güsewell, 2004), or favoring the dominance of graminoids that grow faster under elevated N levels (Demey et al., 2013). As shown by significantly negative correlation between above-ground biomass of *P. kansuensis* and that of graminoids, reduction in plant abundance and growth of *P. kansuensis* following fertilization, the growth of graminoids was increased through the mitigation of parasitism.

Year-to-year variation in the influence by fertilization on plant productivity was found in fertilized and control treatments. The increase in above-ground biomass in control in 2015, following higher early growing season rainfall and average annual temperature, suggested that factors other than fertilization may have impacted on plant biomass production. Indeed, moderate rainfall may enhance fertilization effects (He et al., 2016; You et al., 2017).

### Foliar Nutrient Profiles of Plant Functional Groups

This study is the first to determine the differential effects of fertilization on plant functional groups from a holistic perspective of C, N, and P balances. Differential alteration of foliar nutrient balances among plant functional groups provided a clue for understanding the mechanism underlying the suppression of *P. kansuensis* by fertilization. Among the three foliar nutrient balances tested, the [C | P,N] balance was the only one showing significant effect on plant above-ground biomass. While fertilization influenced the [C | P,N] balance, plants with lower [C | P,N] balance, especially graminoids, benefited more from fertilization in terms of above-ground biomass. Fertilization increased the above-ground biomass of graminoids but reduced that of *P. kansuensis*, the one showed much higher foliar [C | P,N] balance. The occurrence of legumes was too patchy in the study site (with negligible biomass in relative to other functional groups) to allow interpreting the results of tissue nutrient compositions.

Differences in [C | P,N] balances among plant functional groups can be explained by difference in nutrient requirements, acquisition or assimilation (Güsewell, 2004). Foliar concentrations confirmed previous reports that graminoids generally have lower N and P concentrations but higher C assimilation capability than forbs (Güsewell, 2004), accounting for a lower [C | P,N] balance in graminoids. In contrast, higher [C | P,N] balance in *P. kansuensis* than graminoids was due to higher N and P concentrations and lower C concentration, which agreed with a pot cultivation showing higher mineral nutrient concentrations but lower C assimilation and above-ground biomass in *P. kansuensis* than its grass host (Sui et al., 2015).

Inhibited competitiveness of a species in a plant community may be caused by a competitive disadvantage for either below- or above-ground resources (Aerts, 1999). Fertilization significantly increased N and P concentrations in *P. kansuensis*, but little C, suggesting the inhibited competitiveness of *P. kansuensis* could be attributed to reduction in C assimilation rather than mineral nutrient acquisition. The reduced light availability to the root hemiparasite, as shown by increased canopy cover due to vigorous growth of graminoids in fertilized treatments, may have hindered photosynthesis and C assimilation in *P. kansuensis*. In a similar way as non-parasitic plants, root hemiparasites require sufficient light for photosynthesis to transform mineral nutrients into biomass (Matthies, 1995). Although root hemiparasites could extract more heterotrophic organic C from their hosts under shaded conditions, the heterotrophic C may not be sufficient to completely counteract the shading effect (Těšitel et al., 2011). Apart from light competition, higher N supply was found to directly reduce C assimilation in some root hemiparasites. Take *Rhinanthus minor* as an example, much lower concentrations of mannitol (the major soluble carbohydrate for this hemiparasite) were detected in plants grown at a higher N level regardless of its attachment to a host (Jiang et al., 2005). Whether fertilization can directly inhibit C assimilation in *P. kansuensis* remains unknown. Physiological investigations on C assimilation in *P. kansuensis* under controlled conditions separating the effect of soil nutrient availability and that of shading are required to determine if it is shading, high nutrient load, or both, that account for the reduced C assimilation. Furthermore, while it is out of the scope for this study, how the different functional groups maintain their specific nutrient balance across several elements under different fertilization regimes requires further investigations to elucidate species competition in grassland systems. Indeed, the effect of a given factor on plant performance depends on how close to the optimum are other factors (De Wit, 1992).

## Conclusion

The N and P fertilization significantly reduced the growth of *P. kansuensis* and increased the above-ground biomass of the plant community (particularly graminoids). Higher N showed stronger promoting effect on the community productivity and suppressive effect on the root hemiparasite than lower N or P fertilization. However, plant diversity was significantly reduced in HN treatment, compared to other fertilization regimes. Foliar [C | P,N] balance in the plant community correlated negatively with above-ground biomass. While fertilization influenced the [C | P,N] balance, plants with lower [C | P,N] balance, especially graminoids, benefited more from fertilization in terms of above-ground biomass. The inhibited competitiveness of *P. kansuensis*, which showed a much higher [C | P,N] balance, could be attributed to reduced C assimilation rather than mineral nutrient acquisition, as shown by significant increase in foliar N and P concentrations but little increase in C concentration following fertilization. Cultivation experiments addressing physiological processes in C assimilation of *P. kansuensis* under various fertilization and shading regimes are required to unravel the mechanism for reduced C assimilation following fertilization. Furthermore, future investigations could include a larger number of nutrient balances as regulated by fertilization regimes to control the weedy hemiparasite with minimum plant diversity loss.

## Author Contributions

YL, TT, YG, XS, XW, and YH conducted the experiment and collected data. YL, AL, and KG designed the experiment, YL, AL, and S-ÉP analyzed the data. AL wrote and organized the manuscript. All authors contributed to the writing and revising of the manuscript.

## Conflict of Interest Statement

The authors declare that the research was conducted in the absence of any commercial or financial relationships that could be construed as a potential conflict of interest.
